# Efficient algorithms for polyploid haplotype phasing

**DOI:** 10.1186/s12864-018-4464-9

**Published:** 2018-05-09

**Authors:** Dan He, Subrata Saha, Richard Finkers, Laxmi Parida

**Affiliations:** 1 College of Computer Science and Software, Shenzhen University, Shenzhen, 518060 China; 2 IBM T.J. Watson Research Center, 1101 Kitchawan Rd, Yorktown Heights, 10598 NY USA; 3 Wageningen University & Research, 6708 PB, Wageningen, Netherlands

## Abstract

**Background:**

Inference of haplotypes, or the sequence of alleles along the same chromosomes, is a fundamental problem in genetics and is a key component for many analyses including admixture mapping, identifying regions of identity by descent and imputation. Haplotype phasing based on sequencing reads has attracted lots of attentions. Diploid haplotype phasing where the two haplotypes are complimentary have been studied extensively. In this work, we focused on Polyploid haplotype phasing where we aim to phase more than two haplotypes at the same time from sequencing data. The problem is much more complicated as the search space becomes much larger and the haplotypes do not need to be complimentary any more.

**Results:**

We proposed two algorithms, (1) Poly-Harsh, a Gibbs Sampling based algorithm which alternatively samples haplotypes and the read assignments to minimize the mismatches between the reads and the phased haplotypes, (2) An efficient algorithm to concatenate haplotype blocks into contiguous haplotypes.

**Conclusions:**

Our experiments showed that our method is able to improve the quality of the phased haplotypes over the state-of-the-art methods. To our knowledge, our algorithm for haplotype blocks concatenation is the first algorithm that leverages the shared information across multiple individuals to construct contiguous haplotypes. Our experiments showed that it is both efficient and effective.

## Background

Haplotype, or the sequence of alleles residing on the same chromosome, is the fundamental unit of genetic variation. Inference of haplotypes plays an important role in many analyses, including identifying regions of IBD (Identity-by-descent) [1–3], admixture mapping [4], imputation of uncollected genetic variation [5, 6]. Molecular methods [7] are expensive and not amenable to high throughput technologies for obtaining haplotypes. Therefore most studies rely on genotype information and infer haplotypes from genotypes, referred to as haplotype inference or haplotype phasing.

Next-generation sequencing (NGS) technologies have been applied to haplotype phasing as each sequencing read originates from a single chromosome and alleles spanned by that read are on the same haplotype. Phasing diploid haplotypes, especially human haplotypes, has been studied extensively. The human genome is diploid and the two copies of each chromosome are mostly homozygous, namely the alleles at the same positions are mostly identical. However, there are some variations between the pairs and if there are different alleles at the same positions between the pair of chromosomes, they are referred to as heterozygous alleles. For diploid haplotype phasing, only heterozygous alleles are considered and thus the two haplotypes are complimentary to each other.

For diploid haplotype phasing, since many reads overlap with each other, most methods infer haplotypes by partitioning the reads into two sets corresponding to chromosomal origin in such a way that the number of conflicts between the reads and the predicted haplotypes is minimized (such objective function is called Minimum Error Correction, or MEC). Many methods have been proposed for the diploid haplotype phasing problem: HASH [8] and HAPCUT [9] are based on graph structure. He et al. [10] proposed a dynamic programming method as well as a Max-SAT formulation. Deng et al. [11] combined this dynamic programming method with a heuristic approach. Mousavi et al. [12] suggested a HapSat model by converting the haplotype determination problem to a Max-2-SAT problem. HapAssembly converts the haplotype assembly problem to an integer linear programming problem for optimization [13]. Harsh [14] applied a Gibbs Sampling algorithm to phase diploid haplotypes. WhatsHap [15] proposed a fixed parameter tractable approach with coverage as the parameter and can optimize weighted MEC in runtime linear in the number of SNPs.

Recently polyploid haplotype phasing, where more than two haplotypes are phased at the same time, has attracted lots of attention. Polyploid haplotypes mainly come from plant genomes as the plants usually contain more than two haplotypes. Examples of such organisms include potato (which is tetraploid) and wheat (hexaploid). Compared with diploid haplotype phasing, polyploid haplotype phasing is much more challenging as the search space of possible haplotypes increase quadratically with the number of haplotypes. For *k*-haplotypes each with *n* SNPs, the search space is $O\left (2^{(k-1)n}\right)$. Therefore it is very challenging to find the optimal haplotypes.

Various polyploid haplotype phasing methods have been developed. HapCompass [16] relied on a graphical approach to develop a scheme which resolves conflicts arising from incorrect haplotype phasing. HapTree [17] investigated the polyploid setup using a branch-and-bound scheme. SDhaP [18] formulates the haplotype assembly problem as a semi-definite program and exploits its special structure, the low rank of the underlying solution, to solve it rapidly and with high accuracy. H-PoP and H-PoPG [19] conducted Polyploid Balanced Optimal Partition (PBOP).

In this work, we proposed a Gibbs Sampling based algorithm Poly-Harsh. The Gibbs Sampling algorithm considers the polyploid haplotype phasing problem as sampling *k* haplotypes from a huge search space ($O\left (2^{(k-1)n}\right)$, where *k* is the ploidy, *n* is the number of SNPs). The *k* haplotypes minimizes certain objective function (In this work, we take MEC (Minimum Error Correction) as objective function). Poly-Harsh samples the conditional probability of read assignment with fixed haplotypes and haplotype values with fixed read assignment alternatively. We derived a formula for the conditional probability which depends not only on the correct assignment of a read to a haplotype, but also depends on correctly not assigning a read to a haplotype. Our experiments on simulated data showed that the alternative sampling converges fast (usually in less than 100 iterations) and our method achieves a better performance than the state-of-the-art methods.

Haplotype phasing algorithms usually produce blocks of haplotypes due to low coverage or sequencing error. How the blocks should be concatenated is a very challenging problem and has never been resolved. When a single individual is phased, we in general do not have enough information to concatenate the haplotype blocks and the blocks can be only concatenated randomly. However, when a set of individuals inherited from the same founders are phased together, we could leverage the haplotype blocks from all the individuals to better concatenate them. Thus we also proposed an efficient algorithm to concatenate haplotype blocks of a set of individuals from their sequencing data simultaneously. The algorithm consists of three steps (1) candidates generation (2) frequent candidates detection (3) true candidates detection. We showed that our algorithm achieves a much better accuracy than a baseline method, the random concatenation.

## Methods

### Gibbs-sampling

Our method is based on Gibbs sampling and we first introduce the general idea of Gibbs sampling below. Consider the following distribution typically used to perform optimization in graphical models:

1 $$\begin{array}{@{}rcl@{}} P(X) = \frac{1}{Z} exp\left(\mu \sum_{i=1}\sum_{j=1} \phi_{ij}(x_{i}, x_{j})\right) \end{array} $$

where *X*=(*x*_1_,*x*_2_,…,*x*_*d*_) is a *d*-dimensional vector and *Z* is a normalization factor. The function *ϕ* specifies the edge potential for two variables with an edge between them. We would like to collect samples of *X* based on this distribution *P*(*X*).

Gibbs sampler is a special case of Monte Carlo Markov Chain (MCMC) method [20], which is guaranteed to converge to the equilibrium distribution after sufficient burn-in iterations. In each iteration, it randomly samples one variable *x*_*i*_ based on the conditional probability *P*(*x*_*i*_|*x*_[−*i*]_) where all other variables *x*_[−*i*]_=(*x*_1_,*x*_2_,…,*x*_*i*−1_,*x*_*i*+1_,…,*x*_*d*_) are fixed. Formally, this conditional probability can be written based on bayesian rule:

2 $$\begin{array}{@{}rcl@{}} P(x_{i} = t | x_{[-i]}) = \frac{P(x_{i} = t, x_{[-i]})}{\sum_{t'} P(x_{i} = t', x_{[-i]})} \end{array} $$

Readers can refer to [21] for a more detailed description of Monte Carlo Markov Chain.

### Polyploid haplotype phasing

The inputs for the polyploid haplotype phasing problem is the ploidy *k* (the number of haplotypes to be phased), the set of aligned sequencing reads *X* (We assume the raw reads have been aligned to a reference sequence and thus the SNPs spanned by the reads are identified already), a sequencing error rate *ε*. The VCF (Variant Call Format) file containing the SNP positions and dosages could be optional. The dosage information gives the number of reference alleles and alternative alleles for a given SNP position and therefore can be used to reduce the phasing search space and to improve the phasing accuracy. Notice for some programs such as HapCompass, the dosage information is mandatory.

The output of the phasing algorithms is the *k* phased haplotypes. There are a few popular metrics to evaluate the performance of the phasing algorithms, such as MEC (minimum error correction) [22], minimum fragment removal, MSR (minimum single nucleotide polymorphism (SNP) removal) [23], and two recent models MFC (maximum fragments cut) [24] and BOP (balanced optimal partition) [25]. In this work, we focused on MEC as our metric.

### MEC

We focus on minimizing MEC between the phased haplotypes and the input read matrix, which is calculated as the total number of mismatches between the reads and their assigned haplotypes. The following formula is the MEC for polyploid haplotypes:

3 $$\begin{array}{@{}rcl@{}} MEC(X, H) = \sum_{j=1}^{m} \sum_{k=1}^{n} r_{jk} \times D(x_{j}, h_{k}) \end{array} $$

where *X* is a set of *m* sequencing reads, *H* is the set of *n* haplotypes, *x*_*j*_ is the *j*-th read, *h*_*k*_ is the *k*-th haplotype, *D*(*x*_*j*_,*h*_*k*_) is the number of mismatches between *x*_*j*_ and *h*_*k*_,*r*_*jk*_ is 1 if the *j*-the read is assigned to the *k*-the haplotype and 0 vice versa. A read is assigned to a haplotype which minimizes its number of mismatches. Notice mismatches only occur at SNP positions as all other positions are homozygous. Thus the polyploid haplotype phasing problem is to phase *k* haplotypes *H* given the set of reads *X* and the SNP positions so that the objective function *MEC*(*X*,*H*) is minimized. It is known [8] that minimizing MEC is NP-hard even for diploid haplotype phasing when the length of the reads is greater than one.

Recently various methods have been proposed to the polyploid haplotype phasing problem. HapCompass [16] builds a compass graph from the sequencing reads, which is an undirected weighted graph. In the compass graph, the vertices are the SNPs and an edge between a pair of SNPs indicates that at least one read spans the two SNPs. There is an integer weight associated with each edge. It is shown that a compass graph has a unique phasing if it has no conflicting cycles, which is a simple cycle that contains either an odd number of negative edges or at least one 0-weight edge or both. Haplotype phasings correspond to spanning trees in the graph. The phasing problem is converted to a minimum weighted edge removal optimization on the graph and an algorithm based on cycle basis local optimizations for resolving conflicting cycles is proposed.

HapTree [17] aims to trim down the search space for all the possible haplotypes to a much smaller set of more likely solutions. It takes an inductive approach, generating a collection of likely phasing solutions for the first two SNPs in the genome, and then extending those to phasing solutions of the first three SNPs, and those to the first four SNPs, and so on. When extending any particular solution, HapTree chooses (based on computing likelihoods) how the alleles of the newly added SNP may be assigned to chromosomes; it includes only those assignments that are sufficiently likely. Upon including all SNPs to be phased, HapTree randomly chooses a solution of maximum likelihood from amongst the solutions it has found. It is shown [18] that the trimming process might be time consuming for some cases.

SDHaP [18] formulates the haplotype assembly problem as a semi-definite program and exploits its special structure, namely the low rank of the underlying solution, to solve the problem rapidly and with high accuracy. A graph is defined where the nodes are the reads, the edge between two nodes indicate that the two corresponding reads overlap by at least one SNP. A weight is associated with each edge and the weight is computed as the following:

4 $$\begin{array}{@{}rcl@{}} W_{ij} = \frac{k_{sim} - k_{dissim}}{k_{sim} + k_{dissim}} \end{array} $$

where *k*_*sim*_ denotes the number of overlapping positions where the reads have an identical alleles and *k*_*dissim*_ is the number of positions where they are different. Then giving the graph and the ploidy, SDHaP aims to find *k*−1 cuts such that the sum of intra-partition edge weights is maximized and inter-partition edge weights is minimized and the problem is solved via correlation clustering. SDhaP formulates the problem as a semi-definite program (SDP), and employs a low-rank Lagrangian scheme followed by randomized projections and a greedy refinement of the k-ploid haplotypes to solve the SDP.

H-PoP and H-PoPG [19] try to partition the DNA reads sequenced from a *k*-ploid organism into *k* groups such that the reads of the same group share the same alleles on as many SNP loci as possible and the reads from different groups are different on as many loci as possible. Heuristic strategies are proposed by limiting the number of intermediate solutions at each iteration of a dynamic programming algorithm. Notice H-PoP assumes no VCF file, which contains the dosage information of the variants. is provided while H-PoPG accepts VCF file as an input.

The polyploid haplotype phasing method we proposed in this work is an extension of Harsh [14], which applies the Gibbs Sampling algorithm to phase diploid haplotypes (namely two haplotypes). For diploid scenario, the two haplotypes are complimentary. Therefore indeed only one haplotype needs to be phased. For polyploid scenario, the haplotypes are more than two and they can share common segments and are not necessarily complimentary. Thus the problem is much more challenging. Harsh can not be applied to polyploid haplotype phasing in that the conditional probability estimation for the Gibbs Sampling process needs to be completely re-invented.

### Poly-Harsh

#### Haplotype blocks

Notice that there are cases where haplotype components are disconnected, i.e., we need to identify haplotype blocks that are not connected by any reads. There are two possible reasons for disconnected haplotype components, or blocks: the adjacent SNPs might be far from each other, namely their distance is longer than the length of the reads and thus they will not be spanned by any read; the sequencing coverage is low and thus not all SNPs are covered. To identify the haplotype blocks, we can create a graph where the nodes are SNPs and an edge between two SNPs indicates that the two SNPs are connected by some reads. Then we identify the connected components of the graph, which are the SNPs contained in each haplotype block. There would not be any read spanning two blocks and every read only covers the SNPs from a single block. We next phase each block independently, using only the reads covering the SNPs for that specific block.

#### Gibbs sampling

In this work, we developed a Gibbs Sampling based method Poly-Harsh for polyploid haplotype phasing. Our algorithm consists of two major steps: fix the haplotypes, compute read assignments; then fix the read assignments, compute the haplotypes. Here when we compare a haplotype to the reference haplotype, if the allele is the same as the reference, the genotype value is 0. If the allele is alternative, the genotype value is 1. In cases where the allele is neither the reference nor the alternative, we simply assign the genotype value as 1. For each SNP position *i*, we define a genotype value vector *h*_*i*_= [ *g*_1,*i*_,*g*_2,*i*_,…,*g*_*k*,*i*_], where *g*_*j*,*i*_ is the genotype value of the *j*-th haplotype at the *i*-th SNP. For a given ploidy *k* (for illustration purpose and without losing generality, assuming *k* = 4), the genotype value vector could be one of 2^*k*^ binary vectors [1, 0, 0, 0], [1, 1, 0, 0], …, [0, 1, 1, 1]. Notice we ignore vectors [0,0,0,0] and [1,1,1,1] as they indicate the allele is homozygous. We also define a read assignment vector *r* as a *k*-dimensional vector *r*= [ *a*_1_,*a*_2_,…,*a*_*k*_], where *a*_*i*_ = 1 if the read is assigned to the *i*-th haplotype and *a*_*i*_ = 0 if the read is not assigned to the *i*-th haplotype. For example, for *k* = 4, if the read is assigned to the first haplotype, *r*= [ 1,0,0,0]. If the read is assigned to the second haplotype, *r*= [ 0,1,0,0] and so on so forth. Notice a read can not be assigned to more than one haplotype and on the other hand it has to be assigned to one haplotype. Given the genotype value vector *h*_*i*_ for the *i*-th SNP and a read assignment vector *r*_*j*_ for the *j*-th read *x*_*j*_, we define a function as below:

5 $$\begin{array}{@{}rcl@{}} \theta(h_{i}, r_{j}, x_{j}) & = & ln(1-\epsilon)^{t} + ln(\epsilon)^{k-t} \\ t & = & match(h_{i}, r_{j} \times x_{j,i})  \end{array} $$

where *ε* is the sequencing error rate, *x*_*j*,*i*_ is the *i*-th value of the *x*_*j*_,*match*(*A*,*B*) is the vector-wise matches between two vectors *A* and *B* where the number of matches is increased by 1 if two vector elements are identical (either both 1 or both 0). For example, for *h*_*i*_= [ 1,1,0,0], *r*_*j*_=[1,0,0,0],*x*_*j*,*i*_=1, we have *r*_*j*_×*x*_*j*,*i*_= [ 1,0,0,0] and thus *t* =*match*(*h*_*i*_,*r*_*j*_×*x*_*j*,*i*_)= 3. For *h*_*i*_= [ 1,1,0,0],*r*_*j*_=[1,0,0,0],*x*_*j*,*i*_=0, we have *r*_*j*_×*x*_*j*,*i*_=[0,0,0,0] and thus *t* =*match*(*h*_*i*_,*r*_*j*_×*x*_*j*,*i*_)= 2. The *θ* function essentially models the probability of the correct read assignment given the matches between the read and the haplotypes. Notice the function considers the mismatches due to sequencing error.

Given ploidy as *k*, the set of genotype value vectors $H =\, [\!h_{1}, h_{2}, \dots, h_{n}]$ where $h_{i} =\, [g_{1,i}, g_{2,i}, \dots, g_{k_{i}}]$ as we have defined and *n* is the number of SNPs, the set of read assignment vectors $R = \,[\!r_{1}, r_{2}, \dots, r_{n}]$ where $r_{i} = [a_{1}, a_{2}, \dots, a_{k}]$ is the assignment vector for the *i*-th read as we have defined, the sampling process proceeds as follows: We first randomly initiate *H*, then we fix *H* and compute the conditional probability *P*(*R*|*H*). We sample the reads assignment *R* based on *P*(*R*|*H*). Next we fix *R* and compute the conditional probability *P*(*H*|*R*). We sample the genotype value vectors *H* based on *P*(*H*|*R*). For ploidy *k*, we have 2^*k*^ haplotype values for a specific SNP. Assuming the genotype value vector is *h* and the set of reads *X*= [ *x*_1_,*x*_2_,…,*x*_*n*_], we could compute its probability as

6 $$\begin{array}{@{}rcl@{}} P(h | R) = \frac{exp\left(\sum_{j=1}^{n} \theta(h, r_{j}, x_{j})\right)}{exp\left(\sum_{i=1, j=1}^{i=2^{k}, j=n} \theta(h_{i}, r_{j}, x_{j})\right)} \end{array} $$

where the function *θ* is defined in Eq. 5, *h*_*i*_ is the *i*-th genotype value vector, *r*_*j*_ is the assignment vector for the *j*-th read *x*_*j*_. Then we apply sampling to update the genotype values of the SNP. We do a similar Gibbs sampling step for read origin given fixed haplotypes. Again, we conduct sampling to update the read assignment vector on the fixed haplotypes *H* for a given read *x* as below:

7 $$\begin{array}{@{}rcl@{}} P(r | H) = \frac{exp\left(\sum_{j=1}^{2^{k}} \theta(h_{j}, r, x)\right)}{exp\left(\sum_{i=1, j=1}^{i=k, j=2^{k}} \theta(h_{j}, r_{i}, x)\right)} \end{array} $$

where the function *θ* is defined in Eq. 5, *h*_*j*_ is the *j*-th genotype value vector, *r*_*i*_ is the assignment vector when the read *x* is assigned to the *i*-th haplotype.

Given *H* and *R*, we can easily compute the MEC of the phasing. Notice the *k* haplotypes can be constructed from the genotype value vectors *H*, by concatenating all the genotype values from the same haplotype. For example, haplotype one can be constructed as [*g*_1,1_,*g*_1,2_,…,*g*_1,*n*_]. Therefore, the haplotype phasing problem becomes identifying the optimal *H* that minimizes MEC.



Notice for diploid scenario and polyploid scenario, the computations for *P*(*r*|*H*) and *P*(*h*|*R*) are significantly different, as for polyploid scenario, *r* and *h* are *k*-dimensional vectors. The probabilities not only depends on the haplotype the read is assigned to, but also depends on the remaining haplotypes: if one haplotype has a mismatch at a SNP position to a read and the read is not assigned to the haplotype, it is considered as a correct operation to not assign the read to the haplotype.

We repeat the above two steps iteratively. For each iteration, we compute the MEC score of the reads. The process converges when the MEC does not improve or we have reached certain number of iterations. As Gibbs Sampling is sampling based and its performance is affected by the initial random simulation of *H*, it may fall into local optimum solutions. Therefore we re-run the program multiple times, each time started from a different random seed *H*. This helps the program to escape from local optimum. A pseudocode of the algorithm is shown in Algorithm 1. Notice the algorithm shows only one run of the procedure. If we would like to run the algorithm multiple times, we need to repeat steps 1 to 5 multiple times, each time with a different randomly initialized *H*.

## Contiguous haplotype reconstruction

As discussed previously each sample consists of 4 haplotypes. The phasing algorithms may not always produce contiguous sequence of haplotypes for each sample. Instead it produces broken haplotypes due to low coverage depth and/or errors. We can think of the output as blocks of sequences where each block contains contiguous subsequences of 4 broken haplotypes. If the cardinality of blocks is *k* for a particular sample, the number of possible candidates will be 4^*k*^. As there is no prior information available, it is computationally impossible to construct all the 4 haplotypes from 4^*k*^ candidates. Fortunately we have multiple samples and by extracting information from shared haplotypes among samples, we can detect true haplotypes with a very high level of confidence. In this article we propose a randomized algorithm for constructing all 4 haplotypes of each sample.

Here we briefly summarize the 3 fundamental steps of our algorithm. At the beginning for each sample it builds all the candidate haplotypes by concatenating the subsequences in each possible ways from the ordered list of blocks. In the second step the algorithm finds the set of candidate haplotypes which occurs at least twice across the entire set of samples. By utilizing the pruned set of candidate haplotypes, we detect all the 4 haplotypes of each sample. We now describe our algorithm next.

### Generate candidates

At first we recursively construct all possible candidate haplotypes for each sample. Let the number of samples and length of each candidate be *n* and *t*, respectively. Without loss of generality, let each sample consists of *k* blocks of broken haplotypes. If each block contains 4 haplotypes, the number of possible candidates will be 4^*k*^ as stated above. We recursively construct candidates for each sample. The time complexity for constructing all possible candidate haplotypes for *m* samples is $O\left (4^{k}mt\right)$ which is exponential with respect to *k*. It is computationally very expensive task when *k* is large and the execution time can be very large. However in reality *k* is very small and we can safely assume that 1≤*k*≤8.

### Detect frequent candidates

*Frequent candidates* are those candidate haplotypes which occurs multiple times (i.e., at least twice) across *m* samples. This set of candidates contains highly accurate haplotypes because of their multiple occurrences. Exact algorithm is quadratic in the number of candidate haplotypes $\left (\text {i.e}., O\left (\left (4^{k}m\right)^{2}t\right)\right)$. To reduce the runtime we are proposing a randomized algorithm. The expected runtime is sub-quadratic in the number of candidate haplotypes and at the same time the algorithm is also highly accurate in computing the set of frequent candidates. At first we illustrate how to find most similar pair of candidates. By naturally extending it, we will describe the process of finding the set of frequent candidates. We describe our randomized algorithm next.

Suppose we are given *n* vectors $\hat {b}_{1},\hat {b}_{2},\ldots,\hat {b}_{n}$ each of length *t*. The problem is to find the pair of vectors that are the most similar (i.e., the Hamming distance between them is the smallest). Note that, given two vectors, we can find the Hamming distance between them in *O*(*t*) time. A straight forward algorithm to identify the most correlated pair of vectors takes $O\left (n^{2}t\right)$ time. This algorithm computes the Hamming distance between every pair of vectors. We can achieve a better run time using randomization. We say that the correlation between a pair of strings is *p* if the Hamming distance between them is *t*(1−*p*). Let *p*_1_ be the correlation between the most correlated pair of strings and *p*_2_ be the correlation between the second most correlated pair of strings.

The idea of our algorithm is to iteratively collect pairs of strings that are candidates to be the most correlated. Once we collect enough pairs, we compute the distance between each pair in this collection and output the closest. In each iteration we pick *q* columns randomly. For any vector (or string), the values in these columns can be concatenated to get a *q*-bit integer. We hash the vectors based this integer value. Subsequently, we generate pairs as follows: Consider any bucket in the hash table. If there are *m* vectors in this bucket, then each pair of vectors in this bucket is added as a candidate to a list *C*. There are $O\left (n^{\frac {\text {log} p_{1}}{\text {log} p_{2}}}\text {log}\ n\right)$ iterations in the algorithm. We can show that after $O\left (n^{\frac {\text {log} p_{1}}{\text {log} p_{2}}}\text {log}\ n\right)$ iterations, *C* will have the most correlated pair of bulbs with a high probability (i.e., with a probability of 1−*n*^−*Ω*(1)^). For details readers are refereed to [26].

We can naturally extend the above algorithm to get the frequent candidate haplotypes. In each iteration we compute similarity coefficients for all the the pairs in each hash bucket. The similarity coefficient is defined as $SC = \frac {t - d}{t}$ where *t* is the length of the haplotype and *d* is the Hamming distance between a pair of interest. The more the similarity coefficient, the more similar will be the pair. We retain that pair which has the similarity coefficient ≥ a threshold, *V*. The individual candidate haplotype belonging in each pair is then hashed into a hash map *H*. The keys and values of hash map *H* is the candidate strings and frequency of occurrences, respectively. Finally we sort *H* with respect to its values (i.e., the frequency of occurrences) and output candidates occurring at least twice across *m* samples. For each iteration the expected number of pairs generated is *O*(*n*) as described in [26]. The expected time to build the hash map *H* will be also *O*(*n*) for a particular iteration. As the expected time to resolve a collision (i.e., look up and update) for *H* is *O*(1), the total time spent for all the iterations will be $O\left (n^{1+\frac {\log p_{1}}{\log p_{2}}}\right)$. Since we can sort the map by using any integer sorting algorithm, the expected runtime of this step is $O\left (n^{1+ \epsilon }t\log n\right)$ where *n*=4^*k*^*m* and 0<*ε*<1. We have used fixed number of iterations (i.e., 50) in the experimentations. Let the number of frequent candidates be *s*.

### Detect true candidates

Frequent set of candidates contains all the haplotypes shared twice across the samples. Each sample has 4^*k*^ candidate haplotypes. We need to find 4 haplotypes for each sample. Let *S*^′^ and *S*^″^ be the sets of 4^*k*^ candidate haplotypes of a particular sample and frequent candidates, respectively. Intuitively if we find the closest (i.e., most similar) pair of haplotypes (*c*^′^∈*S*^′^,*c*^″^∈*S*^″^), *c*^′^ will be one of the 4 haplotypes of a particular sample. Haplotypes *c*^′^ and *c*^″^ may be identical but it is not guaranteed. *c*^′^ may not be in the set of frequent candidates due to large number of errors. In this case the closest one (i.e., *c*^″^) from *s* gives us the best possible information to find *c*^′^. Next we illustrate this step algorithmically.

Each sample has 4^*k*^ candidates and the size of pruned set is *s* as described in 3. For each candidate *c*^′^∈*S*^′^ and *c*^″^∈*S*^″^, we compute pair-wise similarity coefficient. We then sort all the pairs (*c*^′^,*c*^″^) with respect to *SC* in non-increasing order and then rank of frequent candidates in non-decreasing order. We traverse the sorted list of pairs and collect first 4 candidate haplotypes *c*^′^ with the following restrictions: (1) No candidate haplotype *c*^′^ will be chosen more than once; and (2) Each subsequence from each block will be used exactly once. If the size of each candidate is *t*, we need $O\left (4^{k}st\right)$ time to compute pair-wise similarity coefficients (since the number such pairs is 4^*k*^*s*). Sorting the list of pairs with respect to similarity score and then by rank of frequent candidate haplotypes will take no more than $O\left (4^{k}s(k+\log s)\right)$ time. After sorting detecting first 4 haplotypes could take $O\left (4^{k}st\right)$ time. In total the time complexity to find 4 candidate haplotypes for *m* samples will be $O\left (4^{k}ms(k+t+\text {log}\ s)\right)$.

## Results and discussion

### Polyploid haplotype phasing

In this work, we focused on phasing relatively short regions such as genes, rather than the whole genome. Our simulation pipeline is as below: We randomly simulate one gene of length 1300bp which contains 30 biallelic SNPs. For tetraploid case (*k* = 4) we simulate four haplotypes for each individual. Then we constructed a VCF for each individual according to their simulated haplotypes. Given the VCF and the haplotypes, we next use Mason [27] to simulate the paired-end reads. Mason is a read simulator software for Illumina, 454 and Sanger reads. Its features include coverage, read length, position specific error rates and base quality values. We feed Mason with the VCF with dosage information and set the error rate as 0.01 across all SNP positions and vary the coverage and the read length as shown in Table 1. Illumina paired-end reads are simulated.

**Table 1:** Different parameters for the simulated data

Parameters	Coverage	Read Length
Parameter Set 1	40	100
Parameter Set 2	80	100
Parameter Set 3	40	200
Parameter Set 4	80	200
Parameter Set 5	100	100

**Fig. 1:**
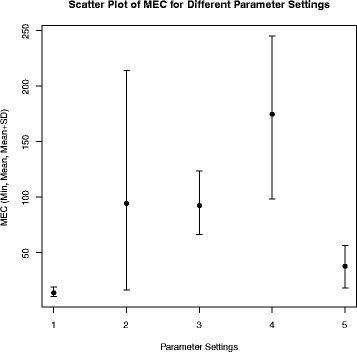
The min, mean and mean+sd of the MEC for Poly-Harsh on 8 rounds of running with respect to different parameter settings

In order to show that re-running Poly-Harsh multiple times in usual leads to better performance as the program could escape from the local optimum, we show in Fig. 1 the minimum, the mean and the standard deviation of 8 rounds of running of Poly-Harsh with respect to each parameter settings. As we can see that for all parameter settings, with 8 rounds of running, the minimum MEC we obtained is in usual much better than the mean MEC, especially when the MEC is relatively large. This indicates that running the program multiple times could in general improve the performance. In our experiments, we see that in general the performance converges in 8 rounds of running. Also we can observe that larger coverage and longer reads lead to larger MEC and larger standard deviation.

**Fig. 2:**
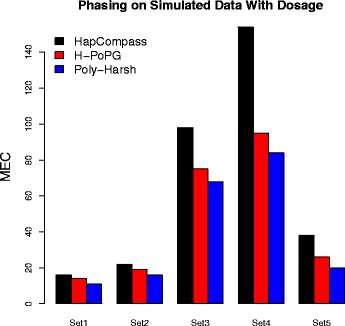
The comparison of MEC for HapCompass, H-PoPG and Poly-Harsh on simulated data with parameter settings specified in Table 1. We feed all methods with the VCF file and dosage information

Next we compare the phasing performance of Poly-Harsh with Hapcompass [16] and H-PoP/H-PoPG [19]. We again take the parameter settings specified in Table 1. For each parameter setting, we randomly simulate 10 data sets and we show the average performance. For Poly-Harsh, we conducted 8 rounds of running, each with 100 iterations. Notice that HapCompass does require the dosage information while both Poly-Harsh and H-PoPG (the version of H-PoP that takes VCF) take the dosage information as optional. Therefore, for a fair comparison, we first feed the dosage information to all three methods. We show the MEC of the three methods in Fig. 2. We can see that both Poly-Harsh and H-PoPG achieved better results compared with HapCompass. Poly-Harsh achieved the best performance for all experiments.

**Fig. 3:**
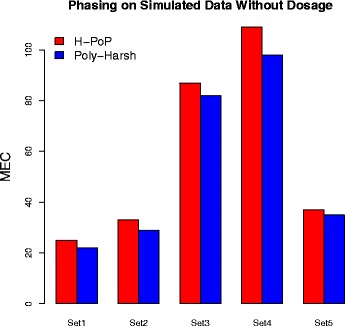
The comparison of MEC for H-PoP and Poly-Harsh on simulated data with parameter settings specified in Table 1. No VCF file is provided

Next we feed only the aligned sequencing reads information to H-PoP and Poly-Harsh for comparison purpose. HapCompass is excluded in the experiments as it requires dosage information. We show that the MEC of both methods in Fig. 3. We can see again Poly-Harsh achieved better results compared with H-PoP. Also the MEC is in general larger when dosage is not provided, indicating that without dosage, the phasing becomes less accurate.

Finally, the execution time of Poly-Harsh depends on the number of rounds of running. For one round of running, on our data sets, the execution time for all three methods are comparable, all less than 1 second. The execution time of Poly-Harsh increases linearly with respect to the number of rounds of running.

### Contiguous haplotype reconstruction

We created 15 simulated datasets each having 30 samples. As described above each sample contains 4 haplotypes. Each haplotype is a binary string of length 1,300 bps. Binary string is created by concatenating randomly chosen binary values (i.e., either 0 or 1) under uniform distribution. As the phased haplotypes usually contain errors, we introduce errors in the haplotypes by flipping bases according to given error rates. For example, if an error rate is 1%, the probability that a base in a haplotype will be flipped is 0.01. Each sample is randomly partitioned into a number of blocks. The number of such blocks will be 1 through 8. As described we are able to construct a haplotype correctly, if it occurs at least twice across the dataset of interest. To make the datasets more realistic, we introduce the concept of *Shared*. If a dataset is told to be *X%* shared, it means *X* haplotypes occurs 2× across all the 30 samples.

We measure the effectiveness of our proposed algorithm using two different metrics. These metrics are defined below.

1. **Sensitivity:** The fraction of the haplotypes correctly constructed. Let the number of constructed and true haplotypes be *P* and *T*, respectively. Now, the Sensitivity can be written as $SN = \frac {P}{T}$
2. **Time:** Measured elapsed time using total number of CPU clock cycles consumed by each of the algorithm.

At first consider datasets D1-D5 (please, see Table 2). These datasets are generated in a way such that each haplotype must occur 2× across the samples. By varying the error rate we measure the performance of our algorithm. For error rate 1−2*%* our algorithm could able to correctly construct all the haplotypes from the set of broken subsequences. For the high error rate (such as, 10% or 20%), it correctly constructs 96.7*%* haplotypes.

**Table 2:** Performance evaluations by varying *shared* and *error rates*. The length of each haplotype is 1,300 bp. Each dataset contain 30 samples. Each sample contains contiguous subsequence of 4 broken haplotypes

Dataset	% Shared	% Error rate	% Sensitivity	Time in seconds
D1	100	0	100.00	19.51
D2		1	100.00	22.02
D3		5	100.00	25.75
D4		10	96.67	35.13
D5		20	96.67	25.88
D6	80	0	96.67	25.53
D7		1	91.66	25.69
D8		5	91.66	26.20
D9		10	94.16	23.51
D10		20	90.00	12.33
D11	60	0	82.50	23.44
D12		1	76.67	19.45
D13		5	72.71	10.49
D14		10	77.50	9.91
D15		20	71.50	12.04

**Fig. 4:**
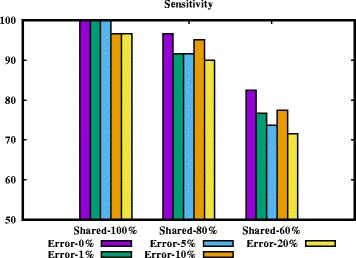
Sensitivity of our algorithm

**Fig. 5:**
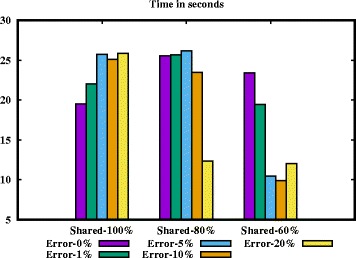
Elapsed time of our algorithm

Now consider datasets D6-D10. 80% haplotypes in each dataset occur twice across the samples. Thus the *sensitivity* should be at least 80%. But the correctness of our algorithm is above 90%. It is due to the fact that if we can construct 3 haplotypes of a sample correctly, the rest will be formed accurately. The same observation applies to datasets D11-D15. We visualize the results in Figs. 4 and 5.

As our method is the very first algorithm that tries to construct contiguous haplotypes from the phased haplotype blocks, we compared our method against a simple baseline method: concatenate the blocks randomly. The baseline method has a sensitivity of around 25% among all scenarios, as the random concatenation doesn’t rely on any information from the dataset. Thus our method is much more accurate than the random concatenation algorithm.

The experimental evaluations show that our algorithm is indeed effective and efficient in terms of both accuracy and runtime. For synthetic dataset, our algorithm achieves nearly 100% accuracy where the datasets have less errors and modest number of shared haplotypes. Here accuracy is defined as the fraction of haplotypes constructed correctly. In some datsets the median of accuracy is 70−80*%*. This is due to the fact that the haplotype construction may be affected if the dataset of interest has very low discriminative power. In this case, there is no sufficient information to distinguish true and false haplotypes accurately. It has cumulative effects also. Each sample has 4 haplotypes as stated above. Suppose we are not able to correctly construct the first haplotype. Then there is a high chance that the rest will be constructed incorrectly. This case arises when (1) dataset contains large number of errors and/or (2) there is little shared information across the samples. In case 2 it is impossible to construct all the haplotypes accurately. In order to construct haplotypes correctly, 3 out of 4 haplotypes of each sample must be occurred at least twice across the samples. In case 1 shared information may be lost because of noise (such as, missing values, phasing errors) in the dataset.

## Conclusion

In this work we proposed a novel polyploid haplotype phasing algorithm that is applicable to any ploidy. The algorithm is based on Gibbs Sampling, where given the set of sequencing reads, we fix the haplotypes and the read assignments alternatively, then estimate the conditional probability of each other and sample their values based on their corresponding conditional probabilities. The Gibbs sampling method has been shown to work well on diploid haplotype phasing [14]. Our experiments illustrate that for tetraploid haplotypes, our method is able to improve the quality of the phased haplotypes (based on Minimum Error Correction) over the state-of-the-art methods. Due to low coverage or sequencing errors, the phased haplotypes usually contain isolated blocks. We proposed an algorithm to construct contiguous haplotypes from the phased haplotype blocks of a set of individuals whose haplotypes are inherited from the same set of founders. To our knowledge, this is the first algorithm that leverages the shared information across multiple individuals to construct contiguous haplotypes. Our experiments showed that our method is both efficient and effective.

Also in our future work, we will evaluate our methods on different metrics like switch error, hamming distance etc. We would also like to evaluate our method on different ploidies to investigate its performance regarding to the number of phased haplotypes.

## Abbreviations

MCMC: Monte carlo markov chain
MEC: Minimum error correction
NGS: Next generation of sequencing
SNP: Single nucleotide polymorphism
VCF: Variant call format
